# Predicting physical activity energy expenditure, types, and intensity using long short-term memory networks in aged 6–18 cross-stage populations

**DOI:** 10.3389/fphys.2026.1852282

**Published:** 2026-07-20

**Authors:** Lei Jiang, Yanan Gao, Chunyu Zhao, Zice Guo, Zhengyuan Huang, Qilin Fan, Junjun Lai, Rufeng Su, Youwei Zhang, Jinxi Zhang

**Affiliations:** 1 Institute of Physical Education and Training, Capital University of Physical Education and Sports, Beijing, China; 2 School of Physical Education and Sport Science, Fujian Normal University, Fuzhou, China; 3 Institute of Artificial Intelligence in Sports, Capital University of Physical Education and Sports, Beijing, China; 4 Department of Exchange and Cooperation, Capital University of Physical Education and Sports, Beijing, China

**Keywords:** adaptive Kalman filter, adolescents, children, energy expenditure, long short-term memory network, physical activity recognition

## Abstract

**Background:**

Accurately estimating physical activity (PA) and energy expenditure of children and adolescents cross-stage are key issues in sports medicine and beyond.

**Objective:**

This study aims to develop and test a long short-term memory (LSTM) network to predict physical activity energy expenditure (PAEE), types, and intensity using accelerometer data collected in children and adolescents.

**Methods:**

A total of 120 healthy participants aged 6–18 years completed 13 PA tasks while wearing triaxial accelerometers at 9 body sites and simultaneously wore the portable metabolic analyzer. 59 features were extracted from accelerometer signals, combined with demographic data to build LSTM models for cross-stage and single-stage (childhood, early and late adolescence), respectively, and compared with the results of 3 ensemble learning (random forest, extreme gradient boosting, and light gradient boosting machine).

**Results:**

The RMSE for predicting PAEE across and single stages were about 0.55 metabolic equivalents (METs) to 0.73 METs, and an average reduction of about 55% in RMSE over ensemble learning. The RMSE for predicting energy expenditure of a specific PA type was averaged about 0.38 METs to 0.49 METs, and an average reduction of about 60% in RMSE over ensemble learning. The recall for predicting PA types was about 90% to 93%, with an average relative improvement of about 70% over ensemble learning. The recall for predicted PA intensity was about 90% to 93%, with an average relative improvement of about 32% over ensemble learning.

**Conclusions:**

LSTM showed strong generalizability and robustness in cross-stage PA monitoring, offering an accurate and scalable solution.

## 1 Introduction

Physical activity (PA) plays a vital role in the growth and development of children and adolescents, prevention of type 2 diabetes and cardiovascular disease risk, weight control, and physical and mental health (Powell et al., 2011; Lister et al., 2023; Perry et al., 2023; Liang et al., 2024). Between the ages of 6 and 18, a critical period of physical growth rapidly, children and adolescents with developmental delays will be at a higher risk of illness, as well as suboptimal motor and cognitive development (Xiong et al., 2023). Considering the complexity and dynamics of individual physiological characteristics at this stage, accurate monitoring of PA intensity and energy expenditure (EE) is critical for assessing energy balance during growth, is the basis for the development of evidence-based exercise intervention programs, and provides critical support for mobile health and sports science (Slade et al., 2021).

Currently, accelerometers are the core tool for PA monitoring due to their objectivity, portability, and cost-effectiveness (Sirard and Pate, 2001). However, the complex nonlinear relationship between raw accelerometer signals and actual EE remains insufficiently characterized, and traditional linear regression-based prediction models have an average prediction error range of approximately 1.6 metabolic equivalents (METs) to 2.1 METs, limiting their applicable aged 6 to 12 (Li et al., 2022; Weaver et al., 2024) or adolescents aged 12 to 18 (Parker et al., 2021) while few have sought to develop unified models spanning the full 6 to 18-year age range cross-stage. This neglects the substantial physiological changes that occur during growth, including shifts in metabolic efficiency and movement economy (Migueles et al., 2017; Liang et al., 2024). Moreover, even within a shared modeling framework, significant performance disparities have been observed across different age stages (Zakeri et al., 2010; Hildebrand et al., 2014; Rosenberger et al., 2016), limiting the broader applicability of these models. Second, many studies have concentrated on a narrow set of physical activities, and model performance tends to deteriorate in multi-class classification tasks involving more than 10 activity types (Swain et al., 2023; Liang et al., 2024). Additionally, the high noise inherent in raw accelerometer signals, coupled with the challenges of processing long temporal sequences, imposes stringent demands on model adaptability and robustness (O’Driscoll et al., 2021; Petersen et al., 2024).

To address these challenges, more advanced modeling strategies are needed. Long short-term memory (LSTM) networks, a specialized form of recurrent neural networks (RNN), are particularly well suited for capturing long-range dependencies in sequential data (Ergen and Kozat, 2018; Sahin and Kozat, 2019; Zhou et al., 2023). The typical RNN often encounters the problem of gradient vanishing and is unable to retain long-term temporal information. In contrast, LSTM adopts a gated memory unit structure, which can effectively store and transmit long-distance temporal features, making it superior to other common deep learning architectures in handling complex accelerometer signals. In this study, we developed the LSTM model capable of handling across stages and multiple PA, based on data collected from triaxial accelerometers and a portable indirect calorimetry system (Houssein et al., 2023), and compared with the results of 3 ensemble learning algorithms: random forest (RF) (Ibrahim et al., 2020), extreme gradient boosting (XGBoost) (Li et al., 2019), and light gradient boosting machine (LightGBM) (Qi et al., 2025). The study pursued 3 primary objectives: (1) to develop and validate a high-accuracy LSTM model for predicting PAEE, activity type, and intensity from accelerometer data in children and adolescents; (2) to compare the prediction accuracy of LSTM and ensemble models across both cross-stage and single-stage settings; and (3) to assess the differences in LSTM model performance across both cross-stage and single-stage prediction tasks.

## 2 Materials and methods

### 2.1 Participants

A total of 120 Chinese primary and secondary school students between the ages of 6 and 18 years cross-stage completed the study, consisting of 3 single-stage including 60 childhood aged 6 to 12 years, 30 early adolescence aged 12 to 15 years, and 30 late adolescents aged 15 to 18 years. The data were collected in March 2023. Each one-year age group comprised 10 participants, with an equal distribution of males and females (5 per sex). Inclusion criteria were: good health, no history of disease, and the ability to safely participate in PA. Exclusion criteria included: presence of organ dysfunction, contraindications to exercise, developmental abnormalities or physical disabilities.

Each participant provided written informed consent. Approval of all ethical and experimental procedures and protocols was granted by the ethical committee of Capital University of Physical Education and Sports under Application No. 2023A036, and performed in line with university requirements.

### 2.2 Data collection and related explanations

#### 2.2.1 Equipment

The instruments used in this study included a triaxial accelerometer, a portable respiratory metabolic monitoring system K5, and a Chinese national standard height meter.

The accelerometer used in this study was manufactured by WitMotion Shenzhen Co. Ltd. (WT901SDCL, China), supports sampling frequencies ranging from 0.1 Hz to 200 Hz, and an acceleration measurement range of ±2/4/8/16 g can be selected. In this study, a sampling frequency of 100Hz per second was used for sampling, and the accelerometers were placed at 9 positions: left and right hips, left and right wrists, left and right thighs, left and right ankles, and chest. Due to variations in sensor sensitivity, device malfunction, or displacement during testing, missing data were observed to varying degrees across all locations. Raw data from all accelerometers were stored locally on SD memory cards and subsequently downloaded to a computer for analysis.

EE and metabolic profiles were assessed using the portable metabolic analyzer COSMED K5 (Rome, Italy) (Kozey et al., 2010). The system-provided METs were used as the primary outcome variable. Additionally, we determined thresholds based on the EE values, which were used to differentiate between exercise intensity levels corresponding to different exercise modes. For participants aged 15 to 18 years, the thresholds were defined as follows: light physical activity (LPA), 1.5 to 3 METs; moderate physical activity (MPA), 3 to 6 METs; and vigorous physical activity (VPA), >6 METs. For participants aged 6 to 15 years, the corresponding thresholds were: LPA, 1.5 to 4 METs; MPA, 4 to 7 METs; and VPA, >7 METs (Rosenberger et al., 2016; Tudor-Locke et al., 2018; Petersen et al., 2020).

#### 2.2.2 Experimental design

Participants were instructed to refrain from engaging in VPA and from consuming caffeine-rich foods or beverages within 48 hours prior to testing. All assessments were scheduled 1.5 to 2 hours postprandially to ensure a stable metabolic state. At the experimental site, participants were required to remain seated at rest for a minimum of 10 minutes before undergoing basic information collection, including date of birth, height, weight, experiment time. The detailed age is determined by the experimental time and the date of birth. Body mass index (BMI) is calculated based on height and weight.

**Figure 1:**
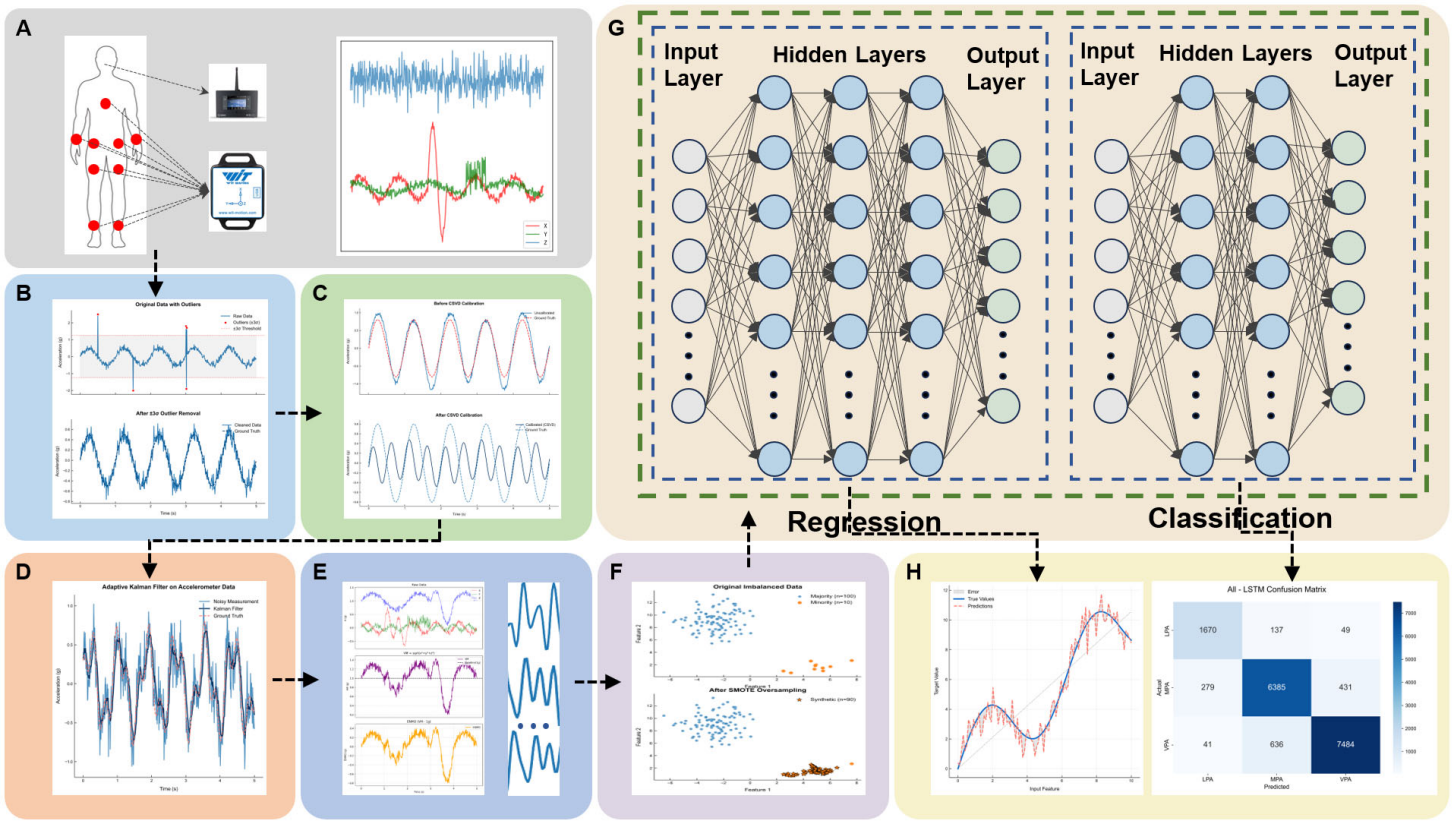
Flow diagram of research. **(A)** Presents the data collected using the K5 device and accelerometer. **(B)** Illustrates the removal of outliers from the data. **(C)** Demonstrates the data calibration process. **(D)** Shows the filtering procedure. **(E)** Describes the computation of vector magnitude and feature extraction. **(F)** Addresses the handling of data imbalance. **(G)** Outlines the LSTM modeling process. **(H)** Displays the results of regression and classification.

Thirteen physical activities comprising 7 different test sections were then performed: slow walking, fast walking, jogging, running, calisthenics, left-legged hopping, right-legged hopping, rope skipping, basketball shooting, basketball dribbling around pole, football dribbling around pole, football passing, and badminton. The experimental site was conducted in a 200-meter indoor basketball court and was completed by the experimenter in a synergistic manner. If participants did not want to or could not continue the test due to reasons such as physical discomfort before or during the test, they stopped immediately. Participants wearing both the triaxial accelerometer and the portable metabolic analyzer COSMED K5 sequentially completed: (1) slow walking, fast walking, jogging, fast running, about 1.5 minutes to 2 minutes each; (2) 1 minute of calisthenics; (3) 30 meters of left-legged hopping and right-legged hopping; (4) 1 minute of rope skipping; (5) 1 minute of self-selected suitable points without retrieving the ball continuous shooting, followed by 2 rounds of cone slalom (8 cones spaced 2 meters apart); (6) 2 round trips of football around the pole between 8 poles (2 meters apart) and 1 minute of football passing with 15 meters spacing; (7) 1 minute of badminton sparring. Rest periods between the 7 test segments were self-paced. Participants resumed the next activity only after confirming full recovery and feeling subjectively ready. **Figure 1** shows the flow of the study design and framework.

#### 2.2.3 Data pre-processing

The preprocessing pipeline consisted of 7 sequential steps: outlier removal, missing data imputation, data calibration, filtering, vector magnitude (VM) computation, feature extraction, and data normalization. Furthermore, this study used a 10-s epoch (Migueles et al., 2017).

##### 2.2.3.1 Outlier removal

Outliers were removed using the ±3σ rule for each acceleration axis direction, as shown in **Figure 1B**.

##### 2.2.3.2 Missing data imputation

As mentioned earlier, due to various factors, there were different degrees of missing data for each part in this study, so only the parts with complete data were retained here, and no processing such as part data filling or symmetrical gap filling was done. The rate of missing data in each part is as follows: left hip (13%), right hip (5%), left hand (8%), right hand (5%), left thigh (5%), right thigh (66%), left foot (28%), right foot (26%), and chest (7%). The data for the right thigh section has a missing rate exceeding 50%. Therefore, the sensor data from this area has been excluded. For the remaining parts, the missing rate is lower than 30%, so they have been selected to be retained.

##### 2.2.3.3 Data calibration

Regarding data calibration, this study used an accelerometer inter-axis orthogonality calibration method based on compact singular value decomposition (CSVD) (He et al., 2020) to effectively solve the problem of inconsistent sensitivity and axial non-orthogonality of tri-axial accelerometers due to the manufacturing process by establishing a data-driven dynamic compensation model, as shown in **Figure 1C**. In addition, a group calibration strategy was adopted to calculate the optimal calibration matrices for different participants, motion modes and body parts, which preserved the specificity of each group of data and ensured the calibration accuracy.

##### 2.2.3.4 Filtering

To address sensor noise, we implemented an optimized AKF (Hesar and Mohebbi, 2021; Wang et al., 2022), which achieves accurate smoothing of motion data by building a six-dimensional state-space model (3D position and 3D velocity), as shown in **Figure 1D** (Chen et al., 2017). We use the before-and-after position change with time difference to estimate the 3D velocity, and adopt the constant velocity model to construct the state transfer matrix, where the position and velocity components are coupled by unit time increments; the observation matrix is designed to only a simplified form that can only detect the position component, which effectively reduces the computational complexity. The key parameters include process noise covariance *Q* (default 0.01) and measurement noise covariance *R* (default 0.1), both initialized as diagonal matrices to establish a complete stochastic modeling framework. At the implementation level, a two-stage iterative process was employed: during the prediction phase, state estimates and covariances were propagated through the transition model; in the update phase, the Kalman gain was computed to optimally weight the measurement residuals.

##### 2.2.3.5 Vector magnitude computation

The VM was calculated for the triaxial accelerometer data and the effect of gravity was removed, was calculated as Euclidean norm minus 1*g* (ENMO) (Migueles et al., 2019; Hibbing et al., 2023) and as described in Equation 1, as shown in **Figure 1E**:

(1) $$\sqrt{x^{2}+y^{2}+z^{2}}-1g$$

where, *x*, *y* and *z* represent the different directions of the accelerometer.

##### 2.2.3.6 Feature extraction

Previous studies used two ways of extracting features for the 3 axes separately and extracting features for the combined vector of the 3 axes (Montoye et al., 2015; Rowlands et al., 2015). In addition, time-domain features alone have been shown to be sufficient for predicting EE and recognizing PA, without the need for frequency-domain features (De Vries et al., 2011). In this study, we integrated both strategies by extracting time-domain features separately from the three individual axes and from the resultant vector magnitude. Building upon prior work, we further expanded the feature set, resulting in a total of 59 time-domain features (Montoye et al., 2015; Rowlands et al., 2015). Details of all the features are shown in **Table 1**.

**Table 1:** Features detail.

Category	Features
Subject features	Gender, age, height, weight, BMI
Acceleration features	X, Y, Z features: minimum, maximum, mean, SD; absolute mean; median crossings; 10th, 25th, 50th, 75th, 90th percentiles; correlations (XY, XZ, YZ); skewness; kurtosis; zero crossing rate Vector magnitude features: minimum, maximum, mean, SD; absolute mean; median crossings; 10th, 25th, 50th, 75th, 90th percentiles; skewness; kurtosis; zero crossing rate
Other features	Acceleration location

##### 2.2.3.7 Data normalization

Considering the consistency of the measure, we used max-min scaler to normalize the data as described in Equation 2 (Chowdhury et al., 2017).

(2) $$X_{std}=\frac{X_{i}-X_{min}}{X_{max}-X_{min}}$$

where, *X_std_* is the normalized data, *X_i_* is the raw data from the accelerometer, *X_min_* is the minimum value in the feature and *X_max_* is the maximum value.

### 2.3 Modeling approach

#### 2.3.1 LSTM principle

This study focuses on the development and testing of LSTM, the various principles of ensemble learning used will not be developed in detail here, and the model parameters are adopted from conventional strategies, only LSTM is introduced here.

LSTM is a state-of-the-art RNN designed to overcome the limitations of standard RNN in capturing long-term dependencies (Sathyanarayana et al., 2016; Hsu et al., 2021; Shalin et al., 2021). LSTM employs a unique architecture that contains input, forgetting, and output gates, as well as a memory cell to control information flow. The forgetting gate decides what information to discard, the input gate updates the cell state with new data, and the output gate determines the next hidden state. This mechanism enables the LSTM to capture long-term dependencies in sequential data, which is effective for tasks that require long retention of context.

#### 2.3.2 LSTM training and testing

According to the previous description, the accuracy rate of high-quality models in predicting the classification of PA types and intensity can exceed 80%, and the RMSE for the regression task of predicting PAEE is about 1 METs, in contrast to the regression task, which has a larger error (4 ± 1 METs), with an error rate of about 25% or more. Based on this, different model architectures are used for the regression and classification tasks respectively.

##### 2.3.2.1 Regression tasks

We developed a 3-layer LSTM architecture for EE prediction across stages of multiple PA. The sliding window is set to time step of 7 and all input features are normalized. By binning and oversampling the target variables to ensure that each time window pattern is fully learned when the sliding window generates sequences, and the continuous EE values are divided into 5 intervals to deal with data imbalance. This part is conducted on the training set. The model architecture and hyperparameters were configured as follows:

The first LSTM layer included 128 units, incorporated both L1 and L2 regularization (λ_1_ = 0.001, λ_2_ = 0.002), and applied 20% input dropout and 15% recurrent dropout to mitigate overfitting; its output sequences were passed to the subsequent layers. Batch normalization was applied after each LSTM layer to stabilize training and accelerate convergence. The second and third LSTM layers contained 96 and 64 units, respectively, and employed L2 regularization (λ = 0.001). A dense layer with 96 Swish-activated units and L2 regularization (λ = 0.001) was added, followed by a 15% dropout layer. This was connected to another Swish-activated dense layer with 64 units. The final output layer consisted of a single neuron with a linear activation function for continuous energy expenditure prediction.

The training process was performed using the Nadam optimizer (initial value of learning rate 0.0005, minimum 1e-6) with logarithmic hyperbolic cosine loss (log-cosh) as the loss function, setting up 200 training cycles (batch size 48), and employing a dynamic learning rate decay (factor=0.5, patience=15) and an early stopping mechanism (patience= 30) to monitor the validation set loss.

##### 2.3.2.2 Classification tasks

We developed a 2-layer LSTM-based classification framework for physical activity classification across multiple age stages. A sliding window with a fixed length of 5-time-step was used to convert the accelerometer sequences into supervised learning samples. The model architecture and hyperparameters were configured as follows:

The first LSTM layer consisted of 128 hidden units and was set to return the full sequence of hidden states to enable temporal context propagation across layers. Both LSTM layers applied a dropout rate of 0.2 to reduce overfitting. The second LSTM layer contained 64 units and passed its final output to a dense layer with 32 ReLU-activated neurons. A dropout layer with a rate of 0.3 was applied after the dense layer for additional regularization. The output layer adopts the Softmax activation function, and the number of neurons strictly corresponds to the number of target categories.

The model was compiled using the Adam optimizer (initial learning rate = 0.001) and trained with categorical cross-entropy loss, a standard objective function for multi-class classification tasks. Early stopping was implemented to prevent overfitting, with a patience of 10 epochs based on validation recall, and the best model weights were restored automatically. The training was conducted for up to 50 epochs using a batch size of 64, and model performance was comprehensively evaluated on the test set.

#### 2.3.3 Ensemble learning training and testing

To ensure both generalizability and predictive accuracy, all ensemble learning algorithms in this study underwent systematic parameter optimization aimed at maximizing model performance. The optimal parameters were determined through iterative refinement, with specific values omitted here for brevity.

#### 2.3.4 Model evaluation

For the regression task of PAEE prediction, model performance was evaluated using the root mean square error (RMSE) and the coefficient of determination (R²).

For the classification tasks involving PA types and intensity, area under the ROC curve (AUC) and confusion matrix were used, and the confusion matrix results were interpreted using recall.

### 2.4 Statistical analysis

We performed all statistical analyses with Python (version 3.9.0) and Tensorflow (version 2.10.0) software. The analytical procedure of this study was implemented strictly following the aforementioned data preprocessing and modeling pipeline. Hyperparameters of all models were determined through multiple rounds of iterative optimization. A participant-level stratified random split was adopted, with participants within each age group randomly divided into training (60%), validation (20%) and test (20%) sets. Each participant and all their collected data were exclusively assigned to a single set. After participant grouping, the accelerometer data were segmented using a 10-s epoch window, followed by the sequential implementation of the pre-processing and modeling procedures described above. Among the entire analytical workflow, outlier removal and data normalization in the preprocessing pipeline were performed only on the training dataset, while the remaining preprocessing steps were applied to the full dataset. The validation set was used exclusively for early stopping during model training, while the test set remained fully independent at the participant level to evaluate generalization performance. All analytical workflows were conducted in strict accordance with standard statistical and machine learning modeling protocols.

## 3 Results

### 3.1 PAEE prediction

#### 3.1.1 Results of PAEE prediction between cross-stage and single-stage populations

**Figure 2:**
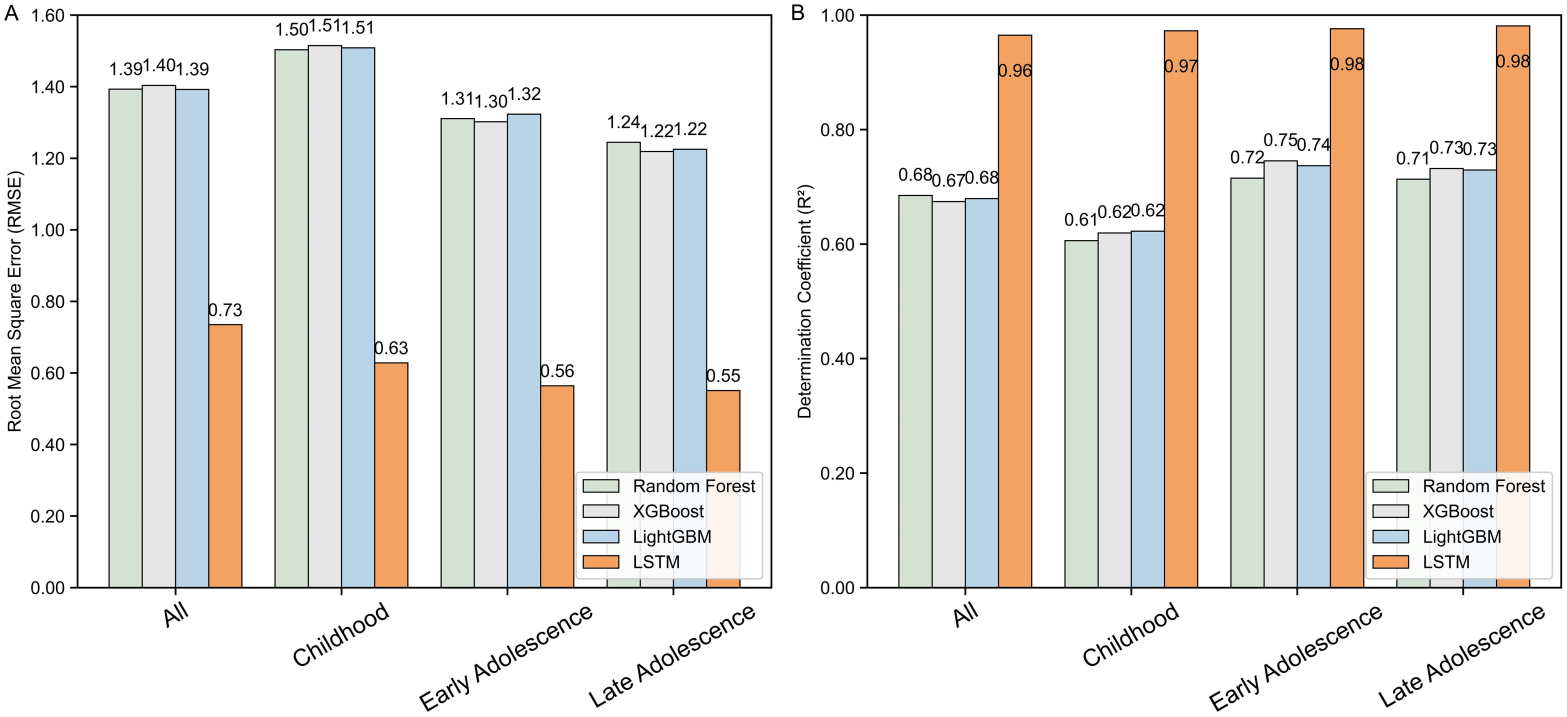
Comparison of PAEE prediction performance between cross-stage and single-stage populations across different modeling methods. **(A)** Root mean square error. **(B)** Coefficient of determination.

**Figure 2** demonstrates the evaluation of the prediction effect of EE under the 4 models cross-stage and single-stage. The 3 ensemble learning models exhibited nearly identical performance across both cross-stage and single-stage scenarios. In contrast, the LSTM model demonstrated a marked improvement over all other methods, with an R² exceeding 0.95, indicating an excellent model fit.

Specifically, the LSTM yielded RMSE of approximately 0.73 METs under the cross-stage, which progressively decreased to 0.63, 0.56, and 0.55 METs across the single-stage. The average RMSE of ensemble learning is about 1.39 METs, 1.51 METs, 1.31 METs, 1.23 METs, respectively. Compared with ensemble methods, the LSTM model achieved an average RMSE reduction of about 55%, with relative decreases of 48%, 58%, 57%, and 55%, respectively.

In terms of the R², the LSTM model achieved values ranging from 0.96 to 0.98 for cross-stage and single-stage, and the ensemble learning model averages about 0.68, 0.62, 0.74, and 0.73, respectively. The LSTM averages a relative improvement of about 41% compared to the ensemble learning, which is about 41%, 56%, 32%, and 34%, respectively.

#### 3.1.2 Results of EE prediction for specific PA type

**Figure 3:**
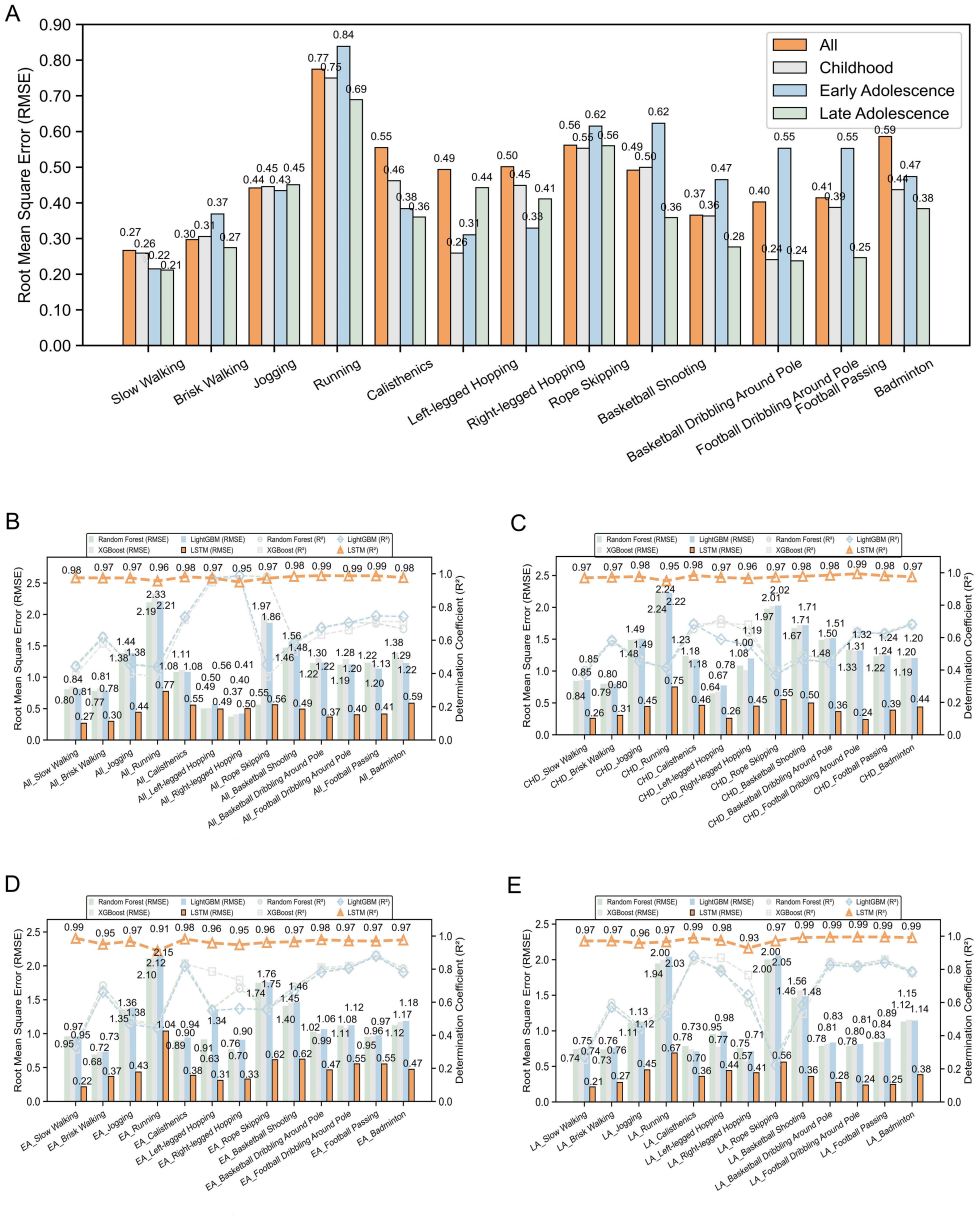
EE prediction results for specific PA type. **(A)** Comparison of RMSE in EE prediction for specific PA type between cross-stage and single-stage populations. **(B)** Comparison of EE prediction performance for specific PA type in cross-stage populations across different modeling methods. **(C)** Comparison of EE prediction performance for specific PA type in childhood across different modeling methods. **(D)** Comparison of EE prediction performance for specific PA type in early adolescence across different modeling methods. **(E)** Comparison of EE prediction performance for specific PA type in late adolescence across different modeling methods. CHD, childhood; EA, early adolescence; LA, late adolescence.

**Figure 3** illustrates the detailed comparison of EE prediction performance for specific PA type using the LSTM model under both cross-stage and single-stage scenarios, alongside results from the 4 modeling approaches across different stage groups.

Overall, LSTM’s EE prediction results for various PA types do not show large differences in RMSE across age stages and are significantly lower than the ensemble learning model. The RMSE under cross-stage and single-stage ranged from about 0.27 METs to 0.77 METs, 0.24 METs to 0.75 METs, 0.22 METs to 1.04 METs, and 0.21 METs to 0.69 METs, with an average of about 0.47 METs, 0.42 METs, 0.49 METs, and 0.38 METs, with only the running part exhibiting relatively high prediction errors. The ensemble learning models did not show significant differences between them, but more importantly, within each stage, large differences were shown in specific PA type. The RMSE ranged from about 0.37 METs to 2.33 METs, 0.66 METs to 2.24 METs, 0.63 METs to 2.15 METs, and 0.57 METs to 2.05 METs under the cross-stage and single-stage scenarios, respectively, which averaged approximately 1.17 METs, 1.33 METs, 1.17 METs, and 1.08 METs, and the 3 components of running, rope skipping, and basketball shooting show higher prediction errors. LSTM reduces about 60% on average when compared to ensemble learning, with reductions of about 68%, 58%, 58%, and 65%, respectively.

The LSTM models were excellent with almost all R² between about 0.95 and 0.99. The ensemble learning models did not show significant differences among them, while the PA types showed large differences among them, with R² of about 0.37 to 0.99, 0.37 to 0.71, 0.32 to 0.88, and 0.22 to 0.88 under cross-stage and single-stage, respectively, with an average of about 0.66, 0.54, 0.65, and 0.64, respectively. The LSTM compared with the ensemble learning, with an average of about 0.66, 0.54, 0.65, and 0.64, respectively, in terms of the relative improvement of about 57%, about 47%, 80%, 49% and 52%, respectively.

### 3.2 Classification of PA types

**Figure 4:**
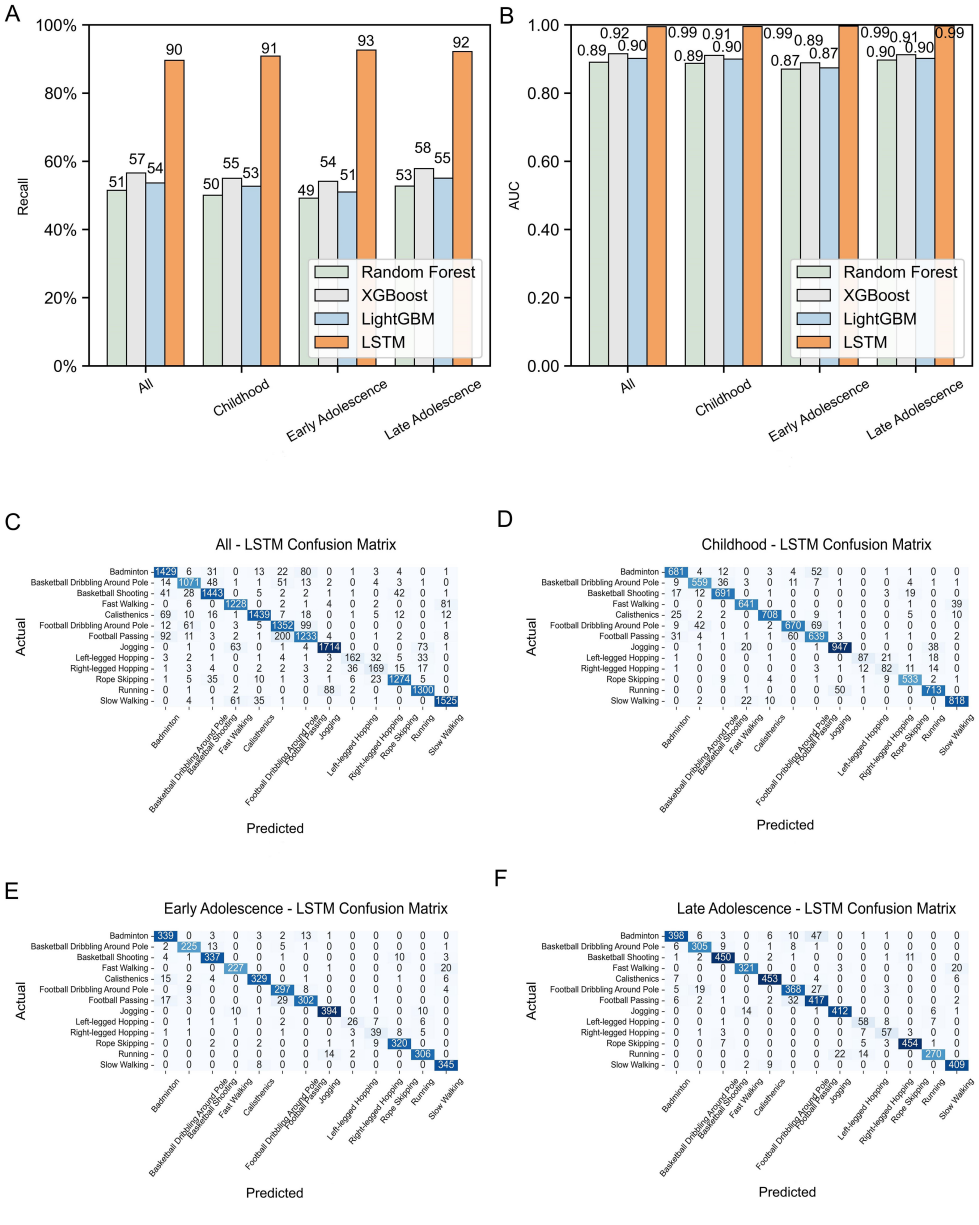
Evaluation of PA types prediction results cross-stage and single-stage. **(A)** Recall. **(B)** AUC. **(C)** Confusion matrix of LSTM-based classification in cross-stage populations. **(D)** Confusion matrix of LSTM-based classification in childhood. **(E)** Confusion matrix of LSTM-based classification in early adolescence. **(F)** Confusion matrix of LSTM-based classification in late adolescence.

**Figure 4** demonstrates the evaluation of the classification results of each model for cross-stage and single-stage PA types. As shown in **Figures 4A, B**, LSTM exhibits excellent classification results compared to ensemble learning, and both are substantially improved, with almost no difference in prediction between cross-stage and single-stage. The LSTM recall is about 90% to 93% under both cross-stage and single-stage. The ensemble learning recall is about 49% to 58% or so, the prediction effect is average, and there is no significant difference in effect between models. Compared with ensemble learning, the relative improvement of classification effect of LSTM model is about 58% to 76%, 65% to 82%, 72% to 90%, 59% to 74%, with an average of about 70%, and the most obvious improvement is in early adolescence; the AUC value under the ROC curve is 0.99, which is close to 1, and the average relative improvement is about 12%, with an average of about 10%, 11%, 14% and 11%, respectively.

The results of the confusion matrices from **Figures 4C–F** show that the recall of the LSTM model under each specific PA type classification result exceeds 90%, except for football dribbling around pole, left-legged hopping and right-legged hopping. This study does not show the confusion matrix results for ensemble learning, but its results show that the recall varies greatly by type, with most PA types having small recall values below 40%.

### 3.3 Classification of PA intensity

**Figure 5:**
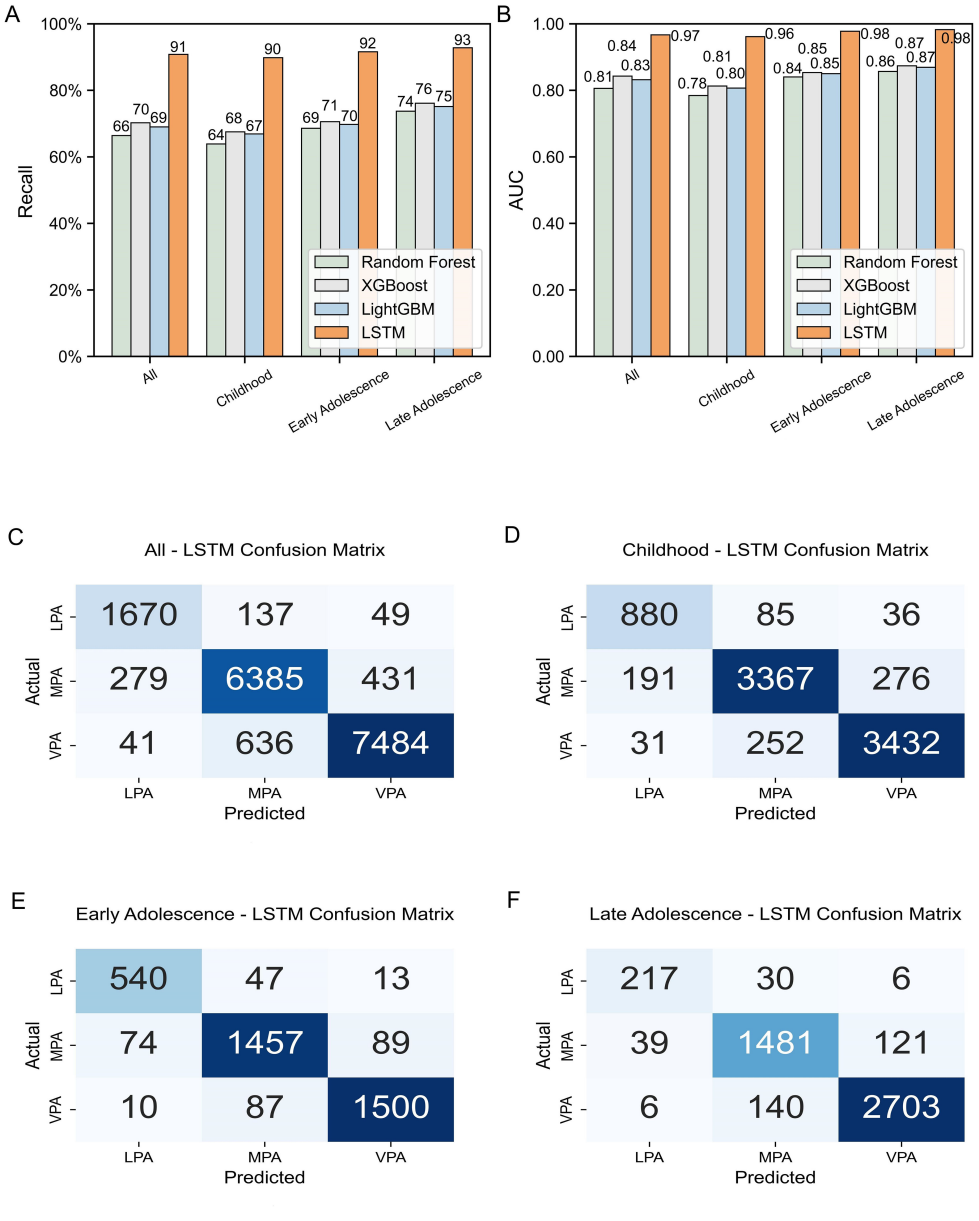
Evaluation of PA intensity prediction results cross-stage and single-stage. **(A)** Recall. **(B)** AUC. **(C)** Confusion matrix of LSTM-based classification in cross-stage populations. **(D)** Confusion matrix of LSTM-based classification in childhood. **(E)** Confusion matrix of LSTM-based classification in early adolescence. **(F)** Confusion matrix of LSTM-based classification in late adolescence.

**Figure 5** demonstrates the evaluation of the classification results of each model in terms of PA intensity across and single-stage. As shown in **Figures 5A, B**, LSTM exhibits excellent classification results compared to ensemble learning, both with some degree of improvement, and there is almost no difference in prediction results between cross-stage and single-stage. The LSTM recall is about 90% to 93% under both cross-stage and single-stage. The ensemble learning recall is about 64% to 76% or so, the prediction effect is moderate, and there is no significant difference in the effect of each model. Compared with ensemble learning, the relative improvement of the classification effect of LSTM model is about 30% to 38%, 32% to 41%, 30% to 33%, and 22% to 26%, with an average of about 32%, and the most obvious improvement for childhood; the AUC value under the ROC curve is 0.96 to 0.98, with an average relative improvement of about 13%, and the average relative improvement is about 19%, 21%, 16%, and 13%, respectively.

The confusion matrix results from **Figures 5C–F** show that the LSTM model has almost recall over 90% for each PA intensity classification result. This study does not show the confusion matrix results for ensemble learning, but its results show that similar to the results for PA types, the recall differences across intensity are also large, mainly in the lower recall for LPA.

## 4 Discussion

### 4.1 Principal findings

To the best of our knowledge, this is the first study to combine AKF and LSTM for the simultaneous prediction of PAEE, types, and intensity across age stages in children and adolescents based on processed accelerometer data. The results of the study confirmed that the model developed in this study achieved excellent predictive results in the cross-stage population. More importantly, except for the slightly higher prediction RMSE for all PAEE, LSTM showed good cross-stage compatibility and robustness for both regression and classification tasks, with no significant difference between the results of cross-stage and single-stage tests respectively, and substantial improvement in prediction accuracy over the ensemble learning. For PAEE prediction, the cross-stage RMSE was reduced by about 55% compared to the ensemble learning for all PA types. reduced by about 55%. Similarly, for specific PA type EE prediction, the cross-stage RMSE was reduced by about 60%. the average relative improvement of recall compared to 3 ensemble learning was about 70% and 32% in the multi-classification task for PA types and intensity, respectively.

### 4.2 PAEE prediction

Although we did not complete the task that there were no significant differences in RMSE between cross-stage and single-stage, the difference in our results has reached a level acceptable to researchers. Most importantly, the regression prediction error has been reduced to some extent compared to current studies on the subject, which is better than almost all current studies. RMSE values for all PA types ranged from about 0.55 METs to 0.73 METs for cross-stage and single-stage, and the R2 ranged from 0.96 to 0.98. Considering that the gap between the RMSE values is 0.1 METs to 0.18 METs and the average EE of PA in this study is about 4 METs, the difference of less than 0.2 METs no longer has an effect, and it can be assumed that the actual predicted outcomes will not be significantly different. Similarly, the mean RMSE values of LSTM across and under a single stage for a given PA types were 0.47 METs, 0.42 METs, 0.49 METs, and 0.38 METs, respectively, and the mean differences did not reach a large magnitude.

In past studies, Montoye et al. (2015) modeled single accelerometer data using ANN with an optimal result prediction of 1.04 METs. Trost et al. (2012) observed RMSE values ranging from 0.9 to 1.1 METs across several neural network models. All of these are higher than the results achieved in our study. When traditional regression-based approaches were applied, prediction errors typically ranged between 1.6 METs and 2.1 METs, further highlighting the advantage of our approach.

In addition, Fuller et al. (2020) studied 9 different wearable devices and showed that none of the commercial devices were accurate at the prediction level of EE. Our results demonstrate this to some extent. The RMSE for PAEE prediction across and single stage was about 1.22 METs to 1.51 METs, and 0.61 METs to 2.33 METs for specific PA type. The larger number of PA types resulted in a larger range of RMSE for ensemble learning for specific PA type compared to previous studies. Although we did not individually adjust this part of the model parameters multiple times, the overall effect was relatively small and the prediction error of PAEE was consistent with previous results.

### 4.3 PA recognition

Our evaluation results show that the predictive recall for both PA types and intensity is about 90% to 93%, which is a high level compared with currently available studies (De Vries et al., 2011; Trost et al., 2012; Hibbing et al., 2018). The predictive recall for PA types and intensity is about 90% to 93%, which is a high level compared with currently available studies. The prediction effects can be considered not significantly different under cross-stage and single-stage.

Particularly, in terms of PA types prediction, since our number of target types reaches 13 and still shows such high accuracy, it is evident that LSTM shows great strength in classification tasks, which also matches LSTM’s own specialization in classification tasks. In addition, in many studies, the prediction accuracy of different PA types may differ by 10% to 20% (De Vries et al., 2011; Trost et al., 2012). Similarly, this study fails to achieve high recall results in the prediction of left-legged and right-legged hopping, indicating that there is still some room for optimization of our model in complex motion scenarios. Single-leg hopping is a high-impact discrete motion characterized by sudden bursts of instantaneous acceleration and intense nonlinear signal fluctuations during the takeoff, flight, and landing phases, which may be related to the difficulty of extracting stable temporal features from fixed accelerometer time windows.

In past studies, for the multiclassification problem, the problem of large differences in the accuracy of activities with different intensity levels often occurs. The results of Hildebrand et al. (2014) mentioned that the accuracy of MPA classification was 33% to 59%, while the accuracy of sedentary and VPA reached more than 90%. And our model well avoids the problem of large prediction differences between different intensity levels. The main reason is that our model parameters are obtained based on many iterations of testing and tuning, and the parameter settings take into account multiclassification, as well as the problem of imbalance between samples.

In our study, ensemble learning showed average and moderate classification results in PA types and intensity, with recalls of 49% to 58% and 64% to 76%, respectively, with significantly lower prediction than LSTM, which may be related to the larger number of categories and the uncertainty of ensemble learning tests. The study by O’Driscoll et al. (2021) mentions that that RF algorithms do not perform better on all wearable devices, with poorer predictions in cross-study validation and also tested algorithms performing at a lower level, which is in line with previous findings and explains to some extent the underperformance of our algorithmic findings in this part of the study. The number of PA types in this study is as high as 13, which also contributes to the low prediction accuracy to some extent, and side-steps the instability of ensemble learning in coping with a larger number of categorization problems.

### 4.4 Pre-processing

The adoption of a more targeted pretreatment process in this study is the basis for excellent generalization ability. Referring to the data preprocessing procedures of previous studies, there is no significant difference in the overall process of this study. The main differences lie in the two parts of data calibration and filtering. In the data calibration section, previous studies mostly adopted SVD decomposition. In contrast, using CSVD can significantly reduce computational complexity, has stronger noise reduction capabilities, and can more smoothly filter out high-frequency noise by truncating tiny singular values. Moreover, it is insensitive to outliers generated by transient impulse types, thereby enhancing robustness (He et al., 2020). For the filtering part, previous studies often adopted low-frequency Extended filters, classical or extended Kalman filter strategies. In this study, the AKF strategy in Kalman filters was used. The main consideration is that the human body posture changes significantly during movement. AKF can adapt to dynamic noise, solve the time-varying problem of accelerometer noise, and can resist the interference of outliers, improve robustness, and avoid the influence of instantaneous errors. It is more efficient in calculation and can be Extended within the Kalman Filter framework, avoiding the complex differentiation of the Extended Kalman Filter (Chen et al., 2017).

### 4.5 Sample imbalance handling

We used split-box oversampling of the data and divided 5 intervals to deal with sample imbalance in regression task, which effectively improved the prediction sensitivity of the model for sparse scenarios such as high-intensity and low-intensity exercise. All EE levels of the data were considered, which effectively improved the generalization ability of the regression task. Few studies have considered and dealt with this issue in past research, which may be one of the reasons for the larger error.

### 4.6 LSTM parameter settings

In the regression task, we construct a 3-layer deep LSTM network with a regularization mechanism, using a 128-96–64 cell-by-layer decreasing architecture. The design effectively suppresses overfitting while maintaining the ability of temporal feature extraction through the synergy of inter-layer batch normalization and hybrid L1/L2 regularization. The combination of “Swish activation function and log-cosh” significantly improves the prediction accuracy of PAEE compared with the traditional “ReLU-Mean Squared Error Loss” scheme.

For PA classification, this study adopted a two-layer LSTM network (128–64 hidden units) coupled with a 5-time-step sliding window to convert raw accelerometer data into time-series inputs. The hierarchical structure captures microscopic motion traits (e.g., gait cycles) and macroscopic activity patterns, effectively reducing sensor noise. The model was trained using the Adam optimizer and categorical cross-entropy loss. The proposed LSTM approach achieved reliable classification performance across age subgroups for PA evaluation.

### 4.7 Developmental physiological basis for cross-age recognition discrepancy

Differences in the development of exercise economy, metabolic scaling, and musculoskeletal maturity during childhood and adolescence limit the general applicability of EE and PA recognition models across different age groups of children. Energy cost may be greater for children due to the greater proportional amount of internal organs in children, their shorter legs, and smaller muscle mass. Puberty increases muscle mass, especially in boys, which could reduce the differences in EE between children and adults (Harrell et al., 2005). As children grow and mature, sex-specific developmental changes in organ weights, organ-specific metabolic rates, muscle mass, and adiposity differentially affect basal metabolic rates and are responsible for the decline in basal metabolic rates (Butte et al., 2018). The motor development and movement patterns differ drastically that younger children display shorter, more intermittent movement bouts with greater postural sway and variability, while adolescents adopt more sustained, adult-like gaits with optimized stride parameters (Voss et al., 2020). Collectively, these developmental shifts alter the mapping between accelerometer-derived features and physiological outcomes, explaining why single-age models fail across age strata and reinforcing the necessity of cross-age compatible modeling to capture dynamic physiological maturation (Pontzer et al., 2021).

### 4.8 Limitations

This study has several limitations that warrant consideration. First, the sample size was relatively limited, with only 10 participants per age group. The total number of participants in both early and late adolescence stages met only the minimal requirements commonly reported in similar studies. Future research should consider increasing the sample size for each age bracket to enhance the robustness of model training and the overall reliability of the findings.

Second, the data were collected under controlled laboratory conditions, and this study did not explore the influence of sensor quantity or placement. These factors may constrain the generalizability of the results in real-world applications and introduce uncertainties regarding practical deployment. Future work could involve data collection in free-living environments and conduct dedicated studies to systematically examine sensor configurations, followed by external validation of the present algorithm under such conditions.

Lastly, the current study did not compare the proposed model with other neural network architectures nor evaluate it on external datasets. Considering that the performance of neural networks is highly sensitive to model structure and hyperparameter tuning, a direct comparison using a unified parameter set may be inappropriate or misleading. As such, we opted for an indirect comparison with results reported in related studies. Recent literature increasingly emphasizes validating models against publicly available benchmark datasets to improve reproducibility and generalizability (Arvidsson et al., 2019). Subsequent studies on the relevant aspects can be carried out in conjunction with other datasets.

### 4.9 Perspective

In conclusion, the findings of this study demonstrate that the model developed by integrating the AKF with LSTM network can accurately predict PAEE, types, and intensity across developmental stages in children and adolescents. The model exhibits strong compatibility and robustness across age groups. In terms of predictive accuracy, it outperforms existing neural network and traditional machine learning algorithms reported in the literature, highlighting its excellent generalization capacity. Its ability to maintain high prediction performance across childhood and adolescents suggests considerable practical value. In the future, it can be attempted to be applied and used on mobile wearable devices to monitor PA in adults and the elderly.

## Data Availability

The datasets presented in this article are not readily available because of individual privacy issues. Requests to access the datasets should be directed to JZ, zhangjinxi@cupes.edu.cn.
